# A rare functional variant of *SHARPIN* attenuates the inflammatory response and associates with increased risk of late-onset Alzheimer’s disease

**DOI:** 10.1186/s10020-019-0090-5

**Published:** 2019-06-20

**Authors:** Yuya Asanomi, Daichi Shigemizu, Akinori Miyashita, Risa Mitsumori, Taiki Mori, Norikazu Hara, Kaoru Ito, Shumpei Niida, Takeshi Ikeuchi, Kouichi Ozaki

**Affiliations:** 1 Division for Genomic Medicine, Medical Genome Center, National Center for Geriatrics and Gerontology, Obu, Aichi Japan; 2 Department of Medical Science Mathematics, Medical Research Institute, Tokyo Medical and Dental University, Tokyo, Japan; 3 Laboratory for Medical Science Mathematics, RIKEN Center for Integrative Medical Sciences, Yokohama, Japan; 4 Department of Molecular Genetics, Brain Research Institute, Niigata University, Niigata, Japan; 5 Laboratory for Cardiovascular Diseases, RIKEN Center for Integrative Medical Sciences, Yokohama, Japan; 6 Medical Genome Center, National Center for Geriatrics and Gerontology, Obu, Aichi Japan

**Keywords:** Alzheimer’s disease, Inflammation, Rare functional variant, Genetic risk factor, *SHARPIN*

## Abstract

**Background:**

Late-onset Alzheimer’s disease (LOAD), the most common form of dementia, results from complicated interactions among multiple environmental and genetic factors. Despite recent advances in genetic analysis of LOAD, more than half of the heritability for the disease remains unclear. Although genetic studies in Caucasians found rare risk variants for LOAD with large effect sizes, these variants are hardly detectable in the Japanese population.

**Methods:**

To identify rare variants possibly explaining part of the genetic architecture for LOAD in Japanese people, we performed whole-exome sequencing analyses of 202 LOAD individuals without the *APOE* ε4 risk allele, a major genetic factor for LOAD susceptibility. We also implemented in vitro functional analyses of the variant(s) to reveal possible functions associated with LOAD risk.

**Results:**

Via step-by-step selection of whole-exome variants, we found seven candidate risk variants. We then conducted a case-control association study in a large Japanese cohort consisting of 4563 cases and 16,459 controls. We finally identified a rare nonsynonymous variant, rs572750141 (NM_030974.3:p.Gly186Arg), in *SHARPIN* that was potentially associated with increased risk of LOAD (corrected *P* = 8.05 × 10^− 5^, odds ratio = 6.1). The amino acid change in SHARPIN resulted in aberrant cellular localization of the variant protein and attenuated the activation of NF-κB, a central mediator of inflammatory and immune responses.

**Conclusions:**

Our work identified a rare functional *SHARPIN* variant as a previously unknown genetic risk factor for LOAD. The functional alteration in SHARPIN induced by the rare coding variant is associated with an attenuated inflammatory/immune response that may promote LOAD development.

**Electronic supplementary material:**

The online version of this article (10.1186/s10020-019-0090-5) contains supplementary material, which is available to authorized users.

## Background

Dementia is one of the leading causes of death in many countries. According to a nationwide survey in 2012, more than 15% of people aged 65 and older in Japan have dementia. (Saji et al., 2019) Currently, there is no effective therapy for LOAD, despite numerous clinical trials of pharmaceutical approaches. Immediate measures against dementia are therefore desired.

The most common form of dementia is Alzheimer’s disease (AD). AD includes the familial form (early-onset AD) and a more common sporadic form (late-onset AD [LOAD]). (Van Cauwenberghe et al., 2016) LOAD results from complex interactions among multiple genetic and environmental factors. (Bagyinszky et al., 2014; Scheltens et al., 2016) A large twin study confirmed that the heritability for AD is high (*h*^2^ = 58–79%). (Gatz et al., 2006) A large-scale genome-wide association study (GWAS) meta-analysis found more than 30 genomic loci associated with the risk of LOAD. (Lambert et al., 2013) However, the *APOE* ε4 allele is the only genetic risk factor for LOAD consistently observed in all races, with the other loci showing smaller effects with modest odds ratios. Accordingly, much of the heritability of LOAD remains unclear.

Recently, two different Caucasian cohort studies identified a rare nonsynonymous variant (rs75932628: p.Arg47His) in *TREM2* as a strong risk factor for LOAD with odds ratio of 2.92 (Jonsson et al., 2013) and 4.59. (Guerreiro et al., 2013) By focusing on this variant, the role of *TREM2* in microglia and its association with LOAD were indicated by using a mouse model of AD. (Wang et al., 2015) However, the minor allele frequency (MAF) of rs75932628 in Japanese LOAD patients was estimated to be just 0.00023 (Miyashita et al., 2014), whereas the population frequency of this variant in Iceland was 0.0063. (Jonsson et al., 2013) Furthermore, in addition to *TREM2*, rare coding variants have been reported in *PLCG2* and *ABI3*. (Sims et al., 2017) However, according to the Genome Aggregation Database (gnomAD, http://gnomad.broadinstitute.org/), none of these variants are found in East Asian individuals. Therefore, we performed a systematic search to identify rare variants that might explain some of the genetic architecture for LOAD in Japanese people.

In this study, we attempted to discover rare genetic variants associated with the risk of LOAD based on whole-exome sequencing analyses of 202 Japanese individuals with LOAD who do not have the *APOE* ε4 risk allele. We identified a rare nonsynonymous variant with strong effect in *SHARPIN*. We also demonstrated that the amino acid substitution of the novel rare risk variant of *SHARPIN* affected its cellular localization and NF-κB activity. Our functional studies of the *SHARPIN* variant thus suggest that this variation promotes the pathogenesis of LOAD by affecting inflammatory/immune pathways.

## Methods

### Study population

Clinical information, which included age, sex, diagnosis, and *APOE* ε4 allele genotype, and the genomic DNA of LOAD patients and normal cognitive function (NC) control participants were obtained from the National Center for Geriatrics and Gerontology (NCGG) Biobank. The NCGG Biobank is one of the facilities belonging to the National Center Biobank Network (NCBN; https://ncbiobank.org/en/home.php). It has been running since 2012. The participants were recruited from NCGG hospital, which is located in Obu city, and the other nearby medical institutes. LOAD was diagnosed at NCGG hospital according to the diagnostic criteria developed by the National Institute on Aging and the Alzheimer’s Association (NIA-AA). (Albert et al., 2011; McKhann et al., 2011) We also applied regional cohort samples of elderly adults (≥ 65 years) in Aichi prefecture, stored in the NCGG Biobank, as general Japanese population samples for the association study. The demographic features of the NCGG samples applied in the exome sequencing and genotyping are shown in Additional file 1: Tables S1–S3. In the first cohort of the association study of two variants, rs572750141 and rs531355933, we also included 7345 DNA samples recruited at RIKEN and public whole-genome sequence data of 3554 Japanese individuals (3.5KJPN, Integrative Japanese Genome Variation; https://ijgvd.megabank.tohoku.ac.jp/) from Tohoku Medical Megabank Organization (TMM).

For the second cohort of the association study of the two variants identified, we obtained 2180 LOAD cases and 2486 controls recruited from Niigata University (Additional file 1: Table S4); their demographics and the clinical criteria used for the diagnosis of AD are described in a previous report. (Miyashita et al., 2014) All individuals are Japanese and provided written informed consent, and the study was performed with the approval of the ethics review board at NCGG and of each institute.

### Exome sequencing and variant calling

For whole-exome sequencing, we used two kinds of next-generation sequencers, a HiSeq 2500 (Illumina, San Diego, CA) and an IonProton (Thermo Fisher Scientific, Waltham, MA). Genomic DNA samples were quantified using the Quant-iT™ PicoGreen® dsDNA Assay Kit or Qubit dsDNA HS Assay Kit (Thermo Fisher Scientific).

For HiSeq, exome libraries were prepared with the Nextera Rapid Capture Expanded Exome Kit (Illumina) or SureSelect Human All Exon V6 (Agilent Technologies, Santa Clara, CA) according to the manufacturers’ protocols. Enriched exome libraries were analyzed by using an Agilent 4200 TapeStation (Agilent) or DNA Fragment Analyzer (Advanced Analytical, Ankeny, IA). The libraries were then sequenced on a HiSeq 2500 system. Paired-end reads of 125 nucleotides were sequenced by using the HiSeq PE Cluster Kit v4 cBot and HiSeq SBS Kit v4. Data processing was performed by using a Resequence/Exome analysis pipeline (Amelieff, Tokyo, Japan). All software in the pipeline was used with properly tuned default settings. Briefly, for use in this pipeline, raw data from the HiSeq system were converted to the FASTQ format with bcl2fastq (Illumina). At the beginning of the pipeline, the FASTQ-formatted files were cleaned up with QCleaner software (Amelieff). Then, sequence reads were mapped to the human genome (hg19) using a BWA algorithm (http://bio-bwa.sourceforge.net/). Duplicated reads were removed by applying Picard (http://broadinstitute.github.io/picard/). Variant calling for single-nucleotide variants (SNVs) and indels was performed with GATK (https://www.broadinstitute.org/gatk/), and variants were outputted in VCF format.

For the IonProton sequencer, exome libraries were prepared with the Ion TargetSeq Exome Enrichment Kit or Ion AmpliSeq Exome RDY Kit (Thermo Fisher Scientific) according to the manufacturer’s protocol. The enriched exome libraries were analyzed by real-time PCR with the GeneRead Library Quant Kit (QIAGEN, Hilden, Germany). Then, libraries were sequenced on an IonProton system, and variants were called by Torrent Suite Software. Variants were outputted in VCF format.

### Annotation

Variants were annotated with dbSNP rs identifiers (rs ID, NCBI dbSNP build 147), allele frequencies, and Combined Annotation Dependent Depletion (CADD) scores (Kircher et al., 2014) by using the ANNOVAR program (http://annovar.openbioinformatics.org/). (Wang et al., 2010) For CADD scores, variants were annotated with CADD version 1.3 (cadd13). For allele frequencies in public databases, we used data from the 1000 Genomes Project (1000g2015aug), Exome Sequencing Project (esp6500siv2_all), and Exome Aggregation Consortium (exac03). We also annotated allele frequencies in the Japanese population by using the 2KJPN database (https://ijgvd.megabank.tohoku.ac.jp/) from TMM.

### Gene expression data

We used gene expression data from the Genotype-Tissue Expression (GTEx) project (https://gtexportal.org/). Median reads per kilobase of exon per million mapped reads (RPKM) data of 13 brain regions from GTEx Analysis V6 were averaged for each gene and used for the variant-filtering step.

### Primers and probes

All primers for PCR reactions, Sanger sequencing, and invader assays were synthesized commercially (Fasmac, Kanagawa, Japan). Primers were designed using the Primer3Plus program (http://primer3plus.com/cgi-bin/dev/primer3plus.cgi).

### Sanger sequencing

PCR was performed using AmpliTaq Gold 360 DNA Polymerase (Thermo Fisher Scientific). Purified PCR fragments were sequenced using the BigDye Terminator v3.1 Cycle Sequencing Kit and an ABI 3100 Genetic Analyzer (Thermo Fisher Scientific).

### Genotyping

We genotyped candidate variants using a multiplex PCR invader assay (Third Wave Technologies, Madison, WI) (Ohnishi et al., 2001) by means of a QuantStudio 7 Flex Real-Time PCR System (Thermo Fisher Scientific) for NCGG and Niigata samples or ABI7900HT Fast Real-Time PCR System (Applied Biosystems) for RIKEN samples. For part of the first cohort control for the two loci (rs572750141 and rs531355933), we also obtained the genotyped data from 1765 in-house whole-genome sequences at RIKEN and the whole-genome sequence data of 3554 Japanese individuals at TMM (3.5KJPN, Integrative Japanese Genome Variation; https://ijgvd.megabank.tohoku.ac.jp/) (Additional file 1: Table S3).

### Construction of plasmid vectors

Each PCR product for wild-type and G186R variant *SHARPIN* was prepared by using complementary DNA synthesized based on mRNA extracted from the buffy coat of the patients analyzed in this study and cloned into pCMV-Myc vector. All inserted sequences were confirmed by Sanger sequencing.

### Luciferase assay

HEK293 cells were transfected with the luciferase reporter plasmid pGL4.32[luc2P/NF-κB-RE/Hygro] (Promega, Madison, WI), and stably expressing cells were selected by hygromycin. Twenty-four hours before transfection, cells were plated on 96-well plates (1.5 × 10^4^ cells/well). Transfection with plasmids was performed using FuGENE® HD Transfection Reagent (Promega). Twenty-four hours after transfection, cells were treated with tumor necrosis factor-α (TNF-α, 20 ng/ml) (Wako, Osaka, Japan) for 5 h. Then, luciferase activity was measured by using a Nano-Glo® Dual-Luciferase® Reporter Assay System (Promega). Each experiment was independently performed three times with five replicates of each sample.

### Immunocytochemistry

HEK293 cells were seeded at a density of 2.0 × 10^4^ cells/well on BioCoat™ Poly-D-Lysine 4-well Culture Slides (Corning, NY, USA), cultured for 24 h, and transfected with wild-type and G186R Myc-*SHARPIN* plasmids. Twenty-four hours after transfection, cells were fixed and incubated with Anti-Myc-tag mAb-Alexa Fluor® 488 (MBL, Nagoya, Japan) according to the manufacturer’s protocol. Then, the slides were mounted with SlowFade™ Diamond Antifade Mountant with DAPI (Thermo Fisher Scientific) and fluorescence images were obtained on a BIOREVO BZ-9000 fluorescence microscope (Keyence, Osaka, Japan).

### Statistical analysis

All statistical analyses were performed using R software (version 3.2.4). For calculation of the odds ratio and 95% confidence interval, vcd package (version 1.4.3) was used in R. Meta-analyses of two cohort sets were performed by using the Mantel-Haenszel χ^2^ test. For luciferase assay experiments, a Student’s t-test was conducted to estimate the statistical difference in luciferase activity among cells transfected with mock (expressing myc), wild-type, and G186R plasmids.

## Results

### Step-by-step filtering to identify rare risk variants

**Fig. 1:**
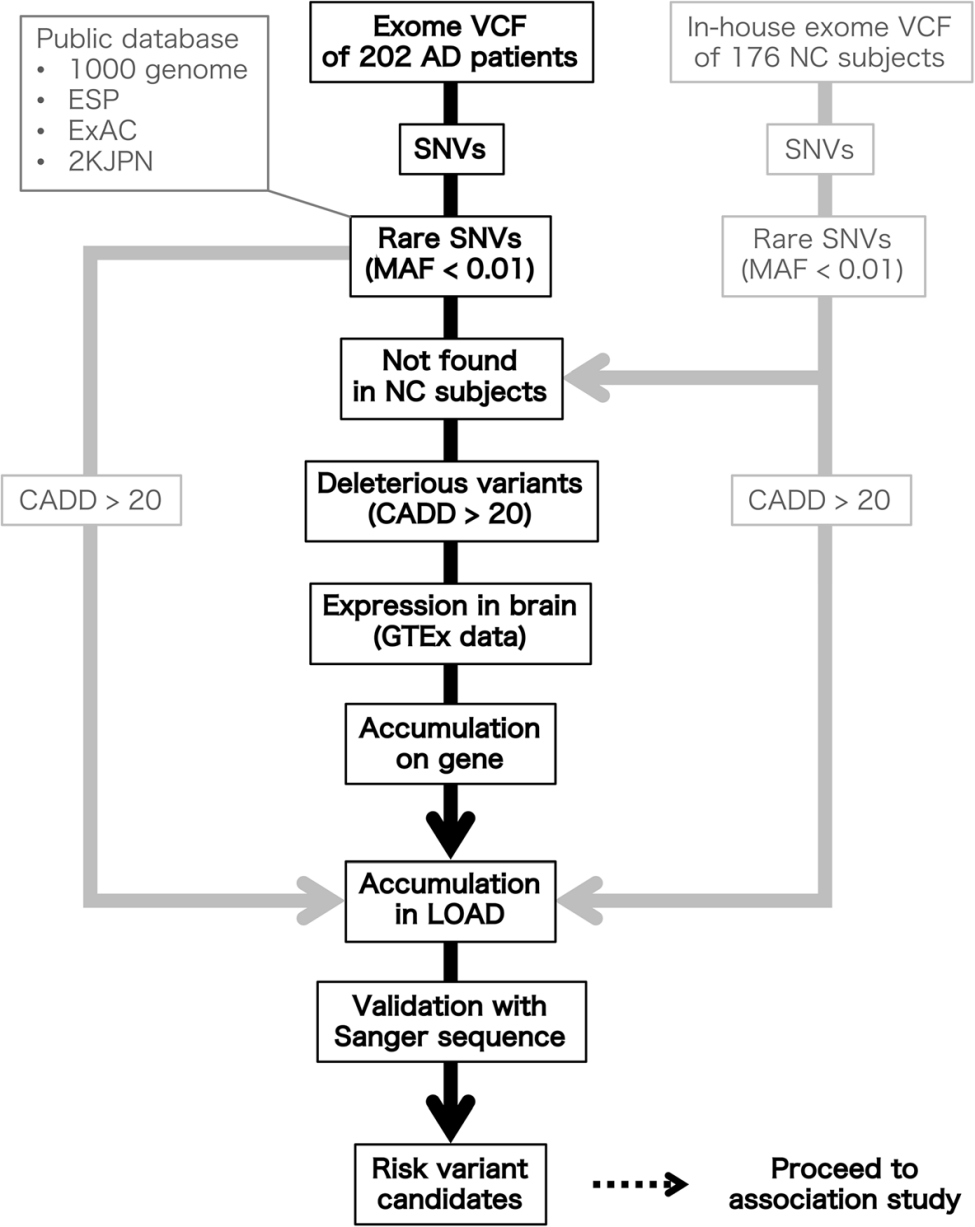
Overview of the risk variant discovery workflow

We performed whole-exome sequencing analyses of 202 LOAD patients lacking the *APOE* ε4 risk allele (Additional file 1: Table S1). In particular, we chose the samples so as to avoid mixed-type dementia (e.g., AD and vascular dementia and AD and dementia with Lewy bodies) as much as possible. All variants derived from exome sequencing analysis were annotated as denoted in the Methods section. First, we checked for known mutations in causal genes—*APP*, *PSEN1*, and *PSEN2*—for autosomal-dominant early-onset AD development. We did not detect any known early-onset AD pathogenic mutations in these genes. Then, all variants of the 202 patients were merged as one VCF-formatted list, which was followed by step-by-step filtering (Fig. 1). Annotated variants were divided into SNVs, indels, and others. In this study, we focused on SNVs because of the accuracy of variant calling. We selected variants with an MAF < 0.01 and those not reported in the annotated allele frequency data of each public database. Then, we excluded the variants found in our own exome sequence data of 176 NC individuals (Additional file 1: Table S1).

The selected rare variants were filtered according to the deleteriousness of the corresponding gene products by means of CADD scores. The distribution of the CADD scores of the variants is shown in Additional file 1: Figure S1. Then, variants with a highly deleterious, scaled C score > 20 were selected. In these steps, we focused on 21,084 variants.

Next, because the brain is an important tissue for AD progression, we excluded genes with low expression in the brain by using expression data from the GTEx database. First, we chose 10,461 genes with 21,084 variants. Then, we selected 8490 genes with an RPKM > 0 and excluded 301 genes with a z-score < − 1.96 (Additional file 1: Figure S2). Ultimately, we chose 8189 genes with 16,837 variants and performed a further filtering step to narrow-down the candidate variants.

We hypothesized that the selected variants of each gene would accumulate against the expected substitution rate and the number of variants of each gene followed a Poisson distribution (Additional file 1: Figure S3). First, we selected 2557 genes with more than two AD patients who had the selected variants. Then, the number of variants of each gene was tested against the expected value from the Poisson distribution function, which was normalized according to CDS length. In this way, 10 genes with significant accumulation, a *q*-value < 0.01 for FDR, were selected, as shown in Additional file 1: Table S5.

Furthermore, the accumulation of variants in LOAD patients against our in-house NC controls was evaluated by using a χ^2^ test. We found a significant accumulation of variants (*q* < 0.05) in four genes: *SHARPIN*, *PXN*, *TYK2*, and *ZNF786* (Additional file 1: Table S6). Then, we confirmed 23 variants in these four genes by using Sanger sequencing; seven variants were non-polymorphic, whereas the others were consistent with exome data. Thus, we selected 16 variants in four genes as the risk factor candidates.

### Association study of candidate variants in the first cohort

To evaluate the associations of the variants found in the exome sequencing analysis with LOAD, we genotyped 16 candidate variants with staged samples, including LOAD cases and NC controls, from the NCGG Biobank. In the first sample set with 1102 cases and 592 controls, nine variants were found in the NC controls, and we further genotyped the other seven variants in an additional 1079 case and 327 control samples. As shown in Table 1, all seven variants were not found in NC controls, whereas *SHARPIN* (rs572750141, p.Gly186Arg) and *TYK2* (rs531355933, p.Val164Met) variants were found in LOAD patients. Then, we assessed the associations of these two variants with LOAD using joint analysis and by increasing the number of samples to 2383 for LOAD cases and to 13,973 for controls (Additional file 1: Table S3). The analysis with Bonferroni correction showed a significant association of the *SHARPIN* variant with LOAD (odds ratio = 8.4) (Table 2).

**Table 1:** Seven candidate risk variants for LOAD

Position (hg19)	Gene	Ref./Alt.	Protein	dbSNP ID	No. of carriers/No. determined	
					LOAD	NC
12:120651694	*PXN*	T/C	p.Tyr487Cys	rs980452538	0/2176	0/909
8:145154709	*SHARPIN*	C/T	p.Gly186Arg	rs572750141	9/2172	0/916
8:145154228	*SHARPIN*	C/T	p.Gly292Arg	rs774802799	0/2183	0/917
19:10477232	*TYK2*	C/T	p.Val164Met	rs531355933	3/2181	0/916
7:148769094	*ZNF786*	C/T	p.Arg257His	rs771874615	0/2179	0/914
7:148767975	*ZNF786*	T/C	p.Tyr630Cys	rs762922242	0/2180	0/915
7:148767897	*ZNF786*	C/T	p.Gly656Asp	NA	0/2175	0/914

NA; Not available

**Table 2:** Associations of the two variants in *SHARPIN* and *TYK2* with the risk of LOAD

dbSNP ID	Chr.	Gene	Phase	Number of samples		Number of variants		MAF		OR	95% CI	*P*	*P* (Bonferroni) ^b^
				Cases	Controls	Cases	Controls	Cases	Controls				
rs572750141	8	*SHARPIN*	1st cohort	2383	13973	10	7	0.002	0.0003	8.4	3.2–22.1	2.28 × 10^−7^	1.6 × 10^−6^
			2nd cohort	2180	2486	1	1	0.0002	0.0002	1.14	0.02–89.5	1	1
			Combined^a^	4563	16459	11	8	0.001	0.0002	6.1	2.4–15.5	1.15 × 10^−5^	8.05 × 10^− 5^
rs531355933	19	*TYK2*	1st cohort	2379	13973	4	5	0.0008	0.0002	4.7	1.3–17.5	0.011	0.77
			2nd cohort	2180	2486	3	4	0.0007	0.0008	0.86	0.13–5.1	1	1
			Combined^a^	4559	16459	7	9	0.0008	0.0003	1.93	0.71–5.2	0.26	1

*ID*; Identifier, *Chr*.; Chromosome, *MAF*; Minor allele frequency, *OR*; Odds ratio, *CI*; Confidence interval

^a^*P* value was calculated by Mantel-Haenszel test

^b^Bonferroni-corrected *P* value, calculated by setting the number of tests to seven

### Replication study and meta-analysis

Next, we implemented a replication study with a second cohort set (2180 LOAD cases and 2486 NC controls) recruited from Niigata University (Additional file 1: Table S4). Carriers of the *SHARPIN* variant were too rare in both case and control groups to evaluate the significance of differences in this cohort (Table 2). However, when we meta-analyzed these data, the *SHARPIN* variant showed a significant risk (corrected *P* = 8.05 × 10^− 5^) with a large effect size (odds ratio of 6.1), both higher than those of the *TREM2* variant in an Icelandic cohort. (Jonsson et al., 2013) The *TYK2* variant showed no significance.

### Functional analysis of the LOAD-associated variant

**Fig. 2:**
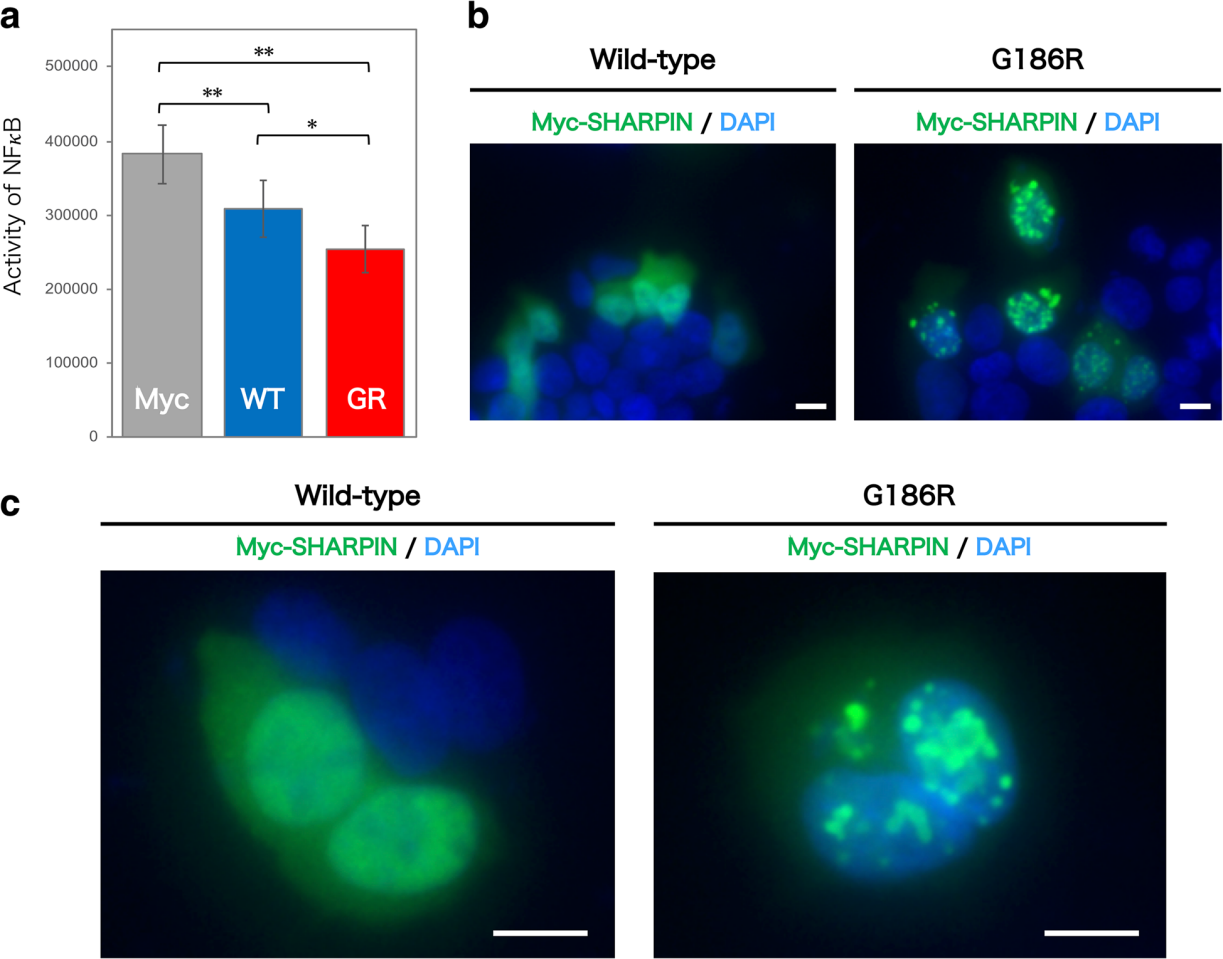
Effect of the G186R variant on SHARPIN function. **a** NF-κB activity in HEK293 cells under TNF-α–induced activation was determined by luciferase assay, which was performed three times with five replicates in each assay. Myc: Myc vector; WT: wild-type Myc-SHARPIN; GR: G186R variant of Myc-SHARPIN. *: *p* < 0.005; **: *p* < 0.0005. **b** Localization of Myc-SHARPIN visualized by immunocytochemistry in HEK293 cells. **c** Magnified images of Myc-SHARPIN. The G186R variant showed granular accumulations of Myc-SHARPIN. Scale bar: 10 μm

To investigate how the LOAD-associated variant found in this study is involved in LOAD pathogenesis, we analyzed the effects of the SHARPIN G186R variant protein. First, because SHARPIN regulates inflammatory and immune responses through activation of the NF-κB pathway, we examined whether the variant protein affects NF-κB activity by means of a luciferase assay. As shown in Fig. 2a, NF-κB activity was significantly decreased when the variant-type SHARPIN—Myc-SHARPIN (G186R)—was expressed in HEK293 cells compared with wild-type Myc-SHARPIN.

We also hypothesized that the amino acid substitution might possibly alter the cellular localization of the protein. Thus, we used immunocytochemistry to examine the cellular localization of SHARPIN, ectopically expressed in HEK293 cells. As shown in Fig. 2b and c and Additional file 1: Figure S4, the G186R variant showed uneven lumps of SHARPIN granules in HEK293 cells, whereas the wild-type was uniformly distributed in the cytosol and had no such accumulations.

## Discussion

We conducted whole-exome sequencing of 202 Japanese LOAD patients without *APOE* ε4 alleles and found a novel coding rare variant (rs572750141, NM_030974.3:p.Gly186Arg) of *SHARPIN* that was significantly associated with a high odds ratio with increased risk of LOAD in the Japanese population (Table 2). However, the results of the association study obtained here have some limitations. Although the meta-analysis showed a significant difference based on sufficient power, only two rs572750141 carriers were found in the second cohort, one case and one control. Therefore, the statistical significance needs to be clarified in another Japanese cohort, and an additional large cohort is required to reduce statistical fluctuations as much as possible. Additionally, our strategy for filtering the exome variants might have some risk gene selection biases due to the step-by-step filtering approach applied. In particular, we focused on genes even slightly expressed in brain tissue. Therefore, genes specifically expressed in peripheral tissues that might nonetheless act as risk factors may have been missed.

SHARPIN is a postsynaptic density protein. (Lim et al., 2001) It is also a component of the linear ubiquitin chain assembly complex (LUBAC). LUBAC regulates inflammation through activation of the NF-κB pathway by conjugating linear ubiquitin chains to IKBKG/NEMO and RIPK1. (Gerlach et al., 2011; Ikeda et al., 2011; Tokunaga et al., 2011) Chronic proliferative dermatitis mutation (cpdm) mice, which are spontaneous *Sharpin*-deficient mutant mice, develop chronic proliferative dermatitis from 3 to 5 weeks of age. (Gallardo Torres et al., 1995; Gijbels et al., 1996; HogenEsch et al., 1993; HogenEsch et al., 1999; Seymour et al., 2006) The lifespan of this mutant mouse is shortened, and the mice show a decreased body size and a characteristic progressive dermatitis with alopecia. In this study, we found that the G186R variant affects the localization of SHARPIN proteins (Fig. 2b and c and Additional file 1: Figure S4) and attenuates TNF-α-induced activation of downstream NF-κB (Fig. 2a). The glycine residue at position 186 of SHARPIN is highly conserved among species (Additional file 1: Figure S5), indicating the importance of this amino acid in cellular function. In fact, this single amino acid substitution drastically altered the cellular localization of SHARPIN. Although it remains to be clarified whether the uneven lumps of SHARPIN G186R granules were due to their uptake into certain organelles or the formation of immunoreactive aggregates, the reduction in NF-κB activity induced by the variant might be involved in this aberrant localization.

In recent studies of rare *TREM2* variants associated with LOAD with large effect sizes, attenuation of the inflammatory response and immune function in the nervous system, particularly in microglia, was reported to contribute to AD onset. (Abbott, 2018; Sims et al., 2017; Wang et al., 2015; Wendeln et al., 2018) SHARPIN also functions as an inflammatory/immune mediator through activation of NF-κB, a central mediator of the inflammatory/immune cascade. Our in vitro experiments with the *SHARPIN* variant revealed its aberrant localization and additional attenuated NF-κB activity, indicating that the variant possibly plays a role in the development of LOAD through aberrant inflammatory/immune functions in the nervous system, which may be similar to the function of the TREM2 variant. Furthermore, a recent computational study indicated the association between the *SHARPIN* gene and AD. (Lancour et al., 2018) In this report, Lancour et al. developed a novel integrated GWAS risk score with a network diffusion approach. They tested this approach on a large GWAS dataset from Caucasians with AD and identified *SHARPIN* as a possible AD risk gene with the second ranked risk score. In addition, a report posted on a preprint server (Soheili-Nezhad et al., 2017) noted that a nonsynonymous variant of *SHARPIN* (rs34173062: p.Ser17Phe) correlated with amygdala atrophy as an endophenotype in AD. Furthermore, RIPK1, which is located downstream of LUBAC, mediates a disease-associated microglial response in AD. (Ofengeim et al., 2017) Taken together, these results suggest that our novel *SHARPIN* variant associates with LOAD onset by affecting inflammation and/or immune function in the nervous system, although the detailed mechanisms underlying the LOAD pathogenesis remain to be clarified.

## Conclusions

We identified a novel coding rare variant of *SHARPIN* as a genetic factor associated with risk of LOAD with a large effect size in the Japanese population via whole-exome sequencing of 202 patients. Our initial sample size is relatively modest for the field of LOAD genetic research in recent years but our pipeline has found a convincing risk factor, indicating the possible appropriateness of our strategy to identify rare disease variants with strong effects. Accordingly, the pipeline applied in this study might be effective for other common diseases. This is the first in vitro demonstration that the rare nonsynonymous variant of *SHARPIN* associated with LOAD onset has critical physiological effects. Further genetic investigations with large East Asian populations may reveal the association between *SHARPIN* and LOAD globally.

Finally, LOAD is a problem not only for patients, but also those that care for them and is also a leading cause of death in many countries. We believe that clarification of the genetic architecture and the implicated pathways helps to explain the pathogenesis of LOAD and will contribute to future medical and pharmaceutical research efforts to develop precision medicine for this common but serious disease.

## Additional file

Additional file 1: **Figure S1.** Distribution of CADD scores. **Figure S2.** Gene expression in the brain. **Figure S3.** Distribution of the number of variants in genes. **Figure S4.** Detailed images of the immunocytochemistry shown in Fig. 2b. **Figure S5.** G186 of SHARPIN is highly conserved among species. Protein sequences of SHARPIN for each species were obtained from UniProt and the region near G186 was compared. **Table S1.** Samples used for exome sequencing. **Table S2.** Demographic features of the NCGG samples genotyped. **Table S3.** Samples used in the first association study of candidate AD risk variants. **Table S4.** Demographic features of the second cohort set used in the association study. **Table S5.** Ten genes with significantly accumulated variants. **Table S6.** Accumulation of variants in LOAD patients compared with NC controls. (PDF 3193 kb)

## Abbreviations

CADD: Combined annotation dependent depletion
gnomAD: Genome Aggregation Database
GTEx: Genotype-Tissue Expression
LOAD: Late-onset Alzheimer’s disease
MAF: Minor allele frequency
NC: Normal cognitive function
NF-κB: Nuclear factor kappa B subunit 1
NIA-AA: National Institute on Aging and the Alzheimer’s Association
RPKM: Reads per kilobase of exon per million mapped reads
SHARPIN: SHANK-associated RH domain interactor
TNF-α: Tumor necrosis factor-α
TREM2: Triggering receptor expressed on myeloid cells 2
