# Supplementary material for: A rare functional variant of SHARPIN attenuates the inflammatory response and associates with increased risk of late-onset Alzheimer’s disease
Source: Mol Med. 2019 Jun 20;25:20. doi: 10.1186/s10020-019-0090-5 (PMC6585023; doi:10.1186/s10020-019-0090-5)
Supplement: Supplementary file 1 — Figure S1. Distribution of CADD scores. Figure S2. Gene expression in the brain. Figure S3. Distribution of the number of variants in genes. Figure S4. Detailed images of the immunocytochemistry shown in Fig. 2b. Figure S5. G186 of SHARPIN is highly conserved among species. Protein sequences of SHARPIN for each species were obtained from UniProt and the region near G186 was compared. Table S1. Samples used for exome sequencing. Table S2. Demographic features of the NCGG samples genotyped. Table S3. Samples used in the first association study of candidate AD risk variants. Table S4. Demographic features of the second cohort set used in the association study. Table S5. Ten genes with significantly accumulated variants. Table S6. Accumulation of variants in LOAD patients compared with NC controls. (PDF 3193 kb) [file 10020_2019_90_MOESM1_ESM.pdf]

**Figure. S1** Distribution of CADD scores.

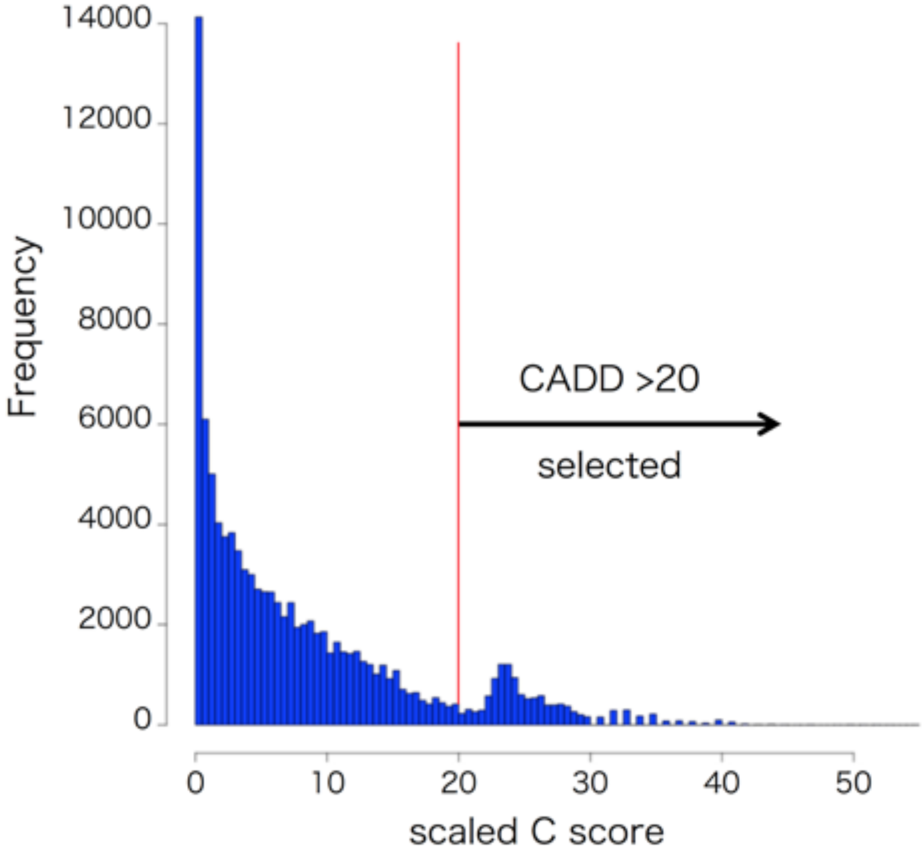

**Figure. S2** Gene expression in the brain.

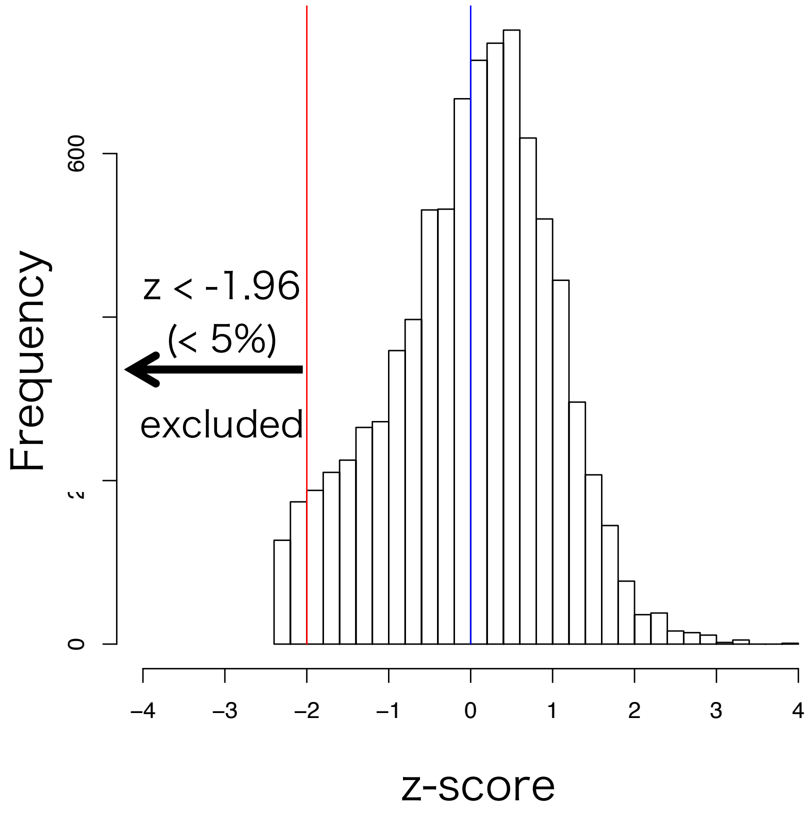

**Figure. S3** Distribution of the number of variants in genes.

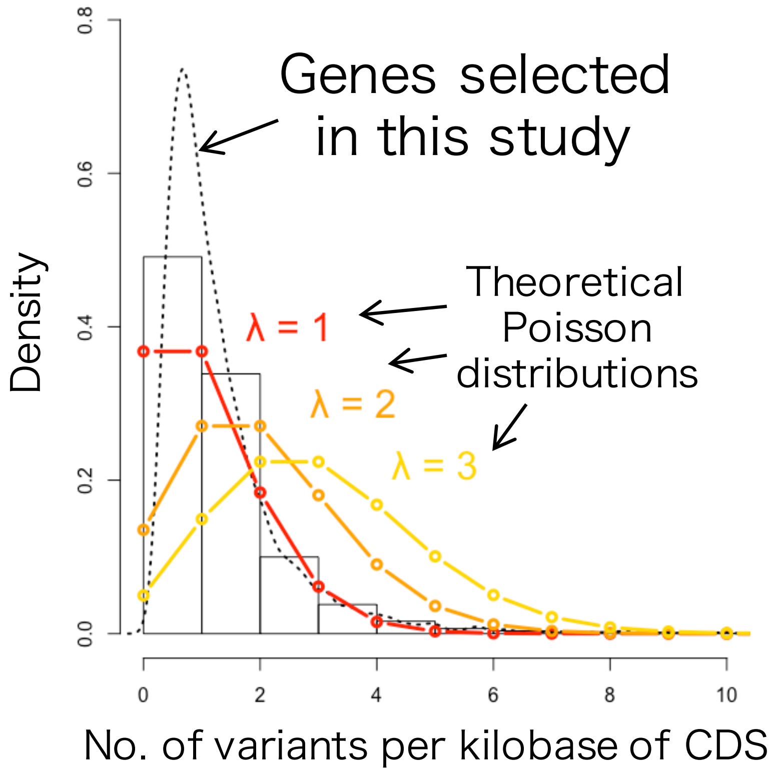

**Figure. S4** Detailed images of the immunocytochemistry shown in Fig. 2b.

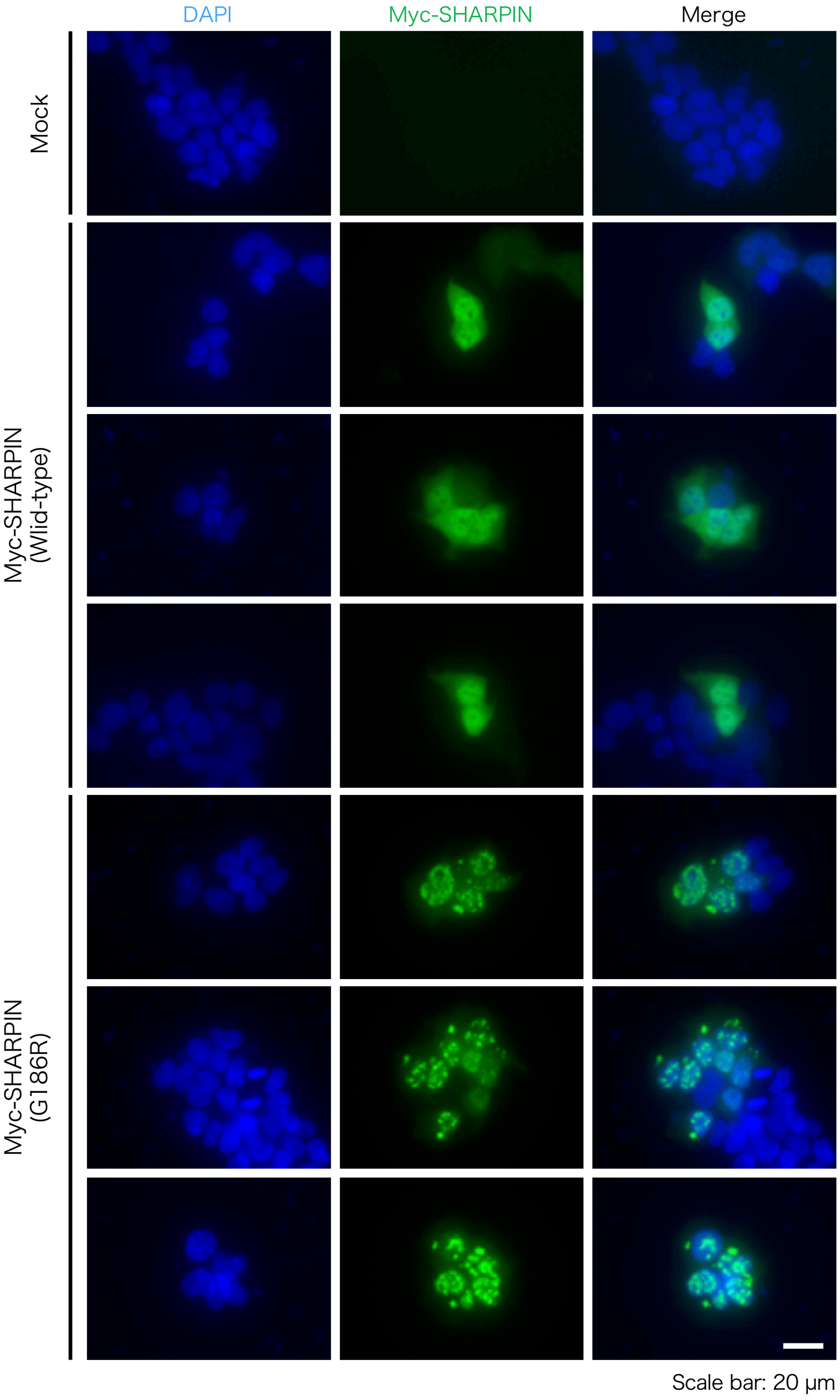

**Figure. S5** G186 of SHARPIN is highly conserved among species. Protein sequences of SHARPIN for each species were obtained from UniProt and the region near G186 was compared.

```
                                                                                   Ubiquitin-like
Species    UniProt_ID                               G186                              domain
Human      SHRPN_HUMAN     167 GNLTEREELAGSLARAIAGGDEKGAAQVAAVLAQHRVALSVQLQEACFPPGPIRLQVTLE 226
Mouse      SHRPN_MOUSE     165 GNF-KKEELATRLSQAIAGGDEKAAAQVAAVLAQHHVALNVQLMEAWFPPGPIRLQVTVE 223
Rat        SHRPN_RAT       165 GNL-KKEELATHLAQAIAGGDEKAAAQVAAILAQHHVALNVQLLEAWFPRGPIRLQVTVE 223
Bovine     SHRPN_BOVIN     165 GDLMEKEELAGRLTRAVEGGDEKGAAQAAAILAQRHVALRVQLQEAYFPPGPIRLQVTVE 224
Pig        F1RSN0_PIG      162 GDLLEKEELAGRLAQAIESGDEKRAAEAAATLAQHHAALRIQLQEACFPPGPVRLQVTVE 221
Dog        J9NZD2_CANLF    156 GDSPEKEELVGRLARAIEDGDEKGAAQAAALLAQHHVALSVRLREACFPPGPIRLQVTVE 215
Cat        M3WFA6_FELCA    162 GDLTEKEELVGRLAQAIEGGDEKGAAQAAATLARHHVALSVQLQEACFPPGPIRLQVTVE 221
Horse      F6VDB9_HORSE    165 GDLMEKEELVGRLARAIEGGDKKGAAQTAAILAQHHVALRVQLQEACFPPGPIRLQVTVE 224
Sheep      W5Q004_SHEEP    165 GDLMEKEELAGRLTQAVEGGDEKGAAQAAAILAQRHVALRVQLQEAYFPPGPIRLQVTVE 224
Bat        G1P7Y4_MYOLU    162 GDLMEKEELAGRLAQAIEDGDGKGAAQAAALLAQHHVALSVQLQESCFPPGPISLQVTVE 221
Alligator  A0A1U8DPC8_ALLSI 44 LELSKTEDLALRLSLAIEVGNEQAASQCAMVLARQQASLRIQPKESSHPTNEISMKVCVE 103
Chameleon  H9GCI4_ANOCA    163 SQLARKEELALRLSAAIDLGDEVEAVRSATALSQQQAPLRIVLKESCYPTSEISMKVQVE 222
Zebrafish  X1WDE8_DANRE    135 LSLSMKEELCVELSRAIEAGDAQAAARYATDLAQQQMTLSIQPAPRDTDDKDISLAVIVE 194
                                .    *:*   *: *:  *:   * . *  *::::  * :           : : * :*
```

**Table S1** Samples used for exome sequencing.

| | | LOAD patients | NC subjects |
|---|---|---|---|
| n | | 202 | 176 |
| Sex | Male | 62 | 99 |
| | Female | 140 | 77 |
| Age : mean (min-max) | | 77.6 (52-94) | 73.2 (65-91) |
| MMSE[a] : mean (min-max) | | 18.5 (0-27) | 29.4 (28-30) |

[a] MMSE: Score of Mini Mental State Examination (MMSE), which is a questionnaire to measure cognitive impairment.

**Table S2** Demographic features of the NCGG samples genotyped.

| | | AD | NC | Total |
|---|---|---|---|---|
| n | | 2,183 | 919 | 3,102 |
| Sex | M | 746 | 453 | 1,199 |
| | F | 1,437 | 466 | 1,903 |
| Age | | 78.7 ± 6.9 | 75.2 ± 6.4 | 77.7 ± 6.9 |
| MMSE | | 18.9 ± 5.4 | 27.6 ± 2.0 | 20.8 ± 6.0 |
| APOE | ε3/3 | 1040 | 582 | 1622 |
| | ε4/3 | 690 | 130 | 820 |
| | ε4/4 | 124 | 13 | 137 |
| | ε3/2 | 90 | 50 | 140 |
| | ε4/2 | 19 | 3 | 22 |
| | ε2/2 | 3 | 3 | 6 |
| | NA | 217 | 138 | 355 |

**Table S3** Samples used in the first association study of candidate AD risk variants.

| Source of Samples | | No. of Samples | |
|---|---|---|---|
| | | Case | Control |
| NCGG | WES | 202 | 176[a] |
| | Genotyping (NCGG hospital) | 2,183 | 919[a] |
| | Genotyping (Elderly cohort) | — | 2,001[a] |
| RIKEN | WGS | — | 1,764 |
| | Genotyping | — | 5,580 |
| TMM | WGS (3.5KJPN) | — | 3,554 |
| Total | | 2,385 | 13,994 |

[a] All individuals were ≥ 65 y/o

**Table S4** Demographic features of the second cohort set used in the association study.

| | | Case | Control | Total |
|---|---|---|---|---|
| n | | 2,180 | 2,486 | 4,666 |
| Sex | M | 651 | 1,363 | 2,014 |
| | F | 1,529 | 1,123 | 2,652 |
| Age | | 78.3 ± 6.1 | 76.3 ± 6.6 | 77.2 ± 6.4 |
| APOE | $\varepsilon$3/3 | 974 | 1,881 | 2,855 |
| | $\varepsilon$4/3 | 921 | 365 | 1,286 |
| | $\varepsilon$4/4 | 194 | 13 | 207 |
| | $\varepsilon$3/2 | 63 | 201 | 264 |
| | $\varepsilon$4/2 | 27 | 20 | 47 |
| | $\varepsilon$2/2 | 1 | 6 | 7 |

**Table S5** Ten genes with significantly accumulated variants.

| Gene Name | Actual No. of variants[a] | Expected No. of variants[b] | *q*-value (FDR) |
|---|---|---|---|
| *MEF2B* | 11 | 1.53 | 0.0017 |
| *ZNF740* | 8 | 0.8 | 0.0027 |
| *TYK2* | 18 | 4.93 | 0.003 |
| *ZNF14* | 13 | 2.67 | 0.003 |
| *DGAT2* | 10 | 1.61 | 0.0033 |
| *SHARPIN* | 10 | 1.61 | 0.0033 |
| *TEKT3* | 11 | 2.04 | 0.0036 |
| *PXN* | 12 | 2.51 | 0.0043 |
| *ROGDI* | 8 | 1.19 | 0.0093 |
| *ZNF786* | 13 | 3.25 | 0.0093 |

[a] Number of filtered-variants in 202 patients

[b] (Total filtered-variants in 202 patients / Total CDS-length of all genes) × CDS-length

**Table S6** Accumulation of variants in LOAD patients compared with NC controls.

| Genes | Carrier in AD | Carrier in NC | Non-carrier in AD | Non-carrier in NC | *q*-value (FDR) |
|---|---|---|---|---|---|
| *SHARPIN* | 13 | 1 | 189 | 175 | 0.025 |
| *ZNF786* | 13 | 1 | 189 | 175 | 0.025 |
| *TKY2* | 26 | 8 | 176 | 168 | 0.025 |
| *PXN* | 12 | 1 | 190 | 175 | 0.025 |
| *DGAT2* | 10 | 1 | 192 | 175 | 0.053 |
| *ROGDI* | 14 | 5 | 188 | 171 | 0.17 |
| *TEKT3* | 23 | 11 | 179 | 165 | 0.17 |
| *MEF2B* | 7 | 2 | 195 | 174 | 0.32 |
| *ZNF740* | 5 | 1 | 197 | 175 | 0.32 |
| *ZNF14* | 15 | 9 | 187 | 167 | 0.48 |
